# Rho kinase inhibition ameliorates cyclophosphamide-induced cystitis in rats

**DOI:** 10.1007/s00210-017-1361-8

**Published:** 2017-02-21

**Authors:** Andrzej Wróbel, Urszula Doboszewska, Ewa Rechberger, Karol Rojek, Anna Serefko, Ewa Poleszak, Krystyna Skalicka-Woźniak, Jarosław Dudka, Piotr Wlaź

**Affiliations:** 1 Second Department of Gynecology, Medical University of Lublin, Jaczewskiego 8, 20-090 Lublin, Poland; 2 Department of Animal Physiology, Institute of Biology and Biochemistry, Faculty of Biology and Biotechnology, Maria Curie-Sklodowska University, Akademicka 19, 20-033 Lublin, Poland; 3 Department of Applied Pharmacy, Medical University of Lublin, Chodźki 1, 20-093 Lublin, Poland; 4 Department of Pharmacognosy with Medicinal Plant Unit, Medical University of Lublin, Chodźki 1, 20-093 Lublin, Poland; 5 Department of Toxicology, Medical University of Lublin, Jaczewskiego 8b, 20-093 Lublin, Poland

**Keywords:** Cyclophosphamide-induced cystitis, Overactive bladder, Bladder inflammation, Cystometry, Rho kinase inhibitor

## Abstract

Hemorrhagic cystitis often develops in patients treated with cyclophosphamide (CYP). Studies have indicated that Rho kinase (ROCK) inhibitors may suppress detrusor overactivity symptoms and possess anti-inflammatory properties. The aim of the present study was to investigate whether inhibition of ROCK reduces cystometric and histopathological changes associated with CYP-induced cystitis. The rats received GSK 269962, a ROCK inhibitor, at a dose of 30 mg/kg daily, or vehicle for 7 days. Then, acute chemical cystitis leading to bladder overactivity was induced by CYP injection (200 mg/kg i.p.). Following CYP injection, cystometric studies with physiological saline were performed. Moreover, bladder edema (by the Evans Blue dye leakage technique) and urothelium thickness were measured. CYP injection resulted in a significant increase in cystometric parameters: basal pressure, threshold pressure, bladder contraction duration, relaxation time, detrusor overactivity index, non-voiding contractions amplitude, and non-voiding contractions frequency as well as increased Evans Blue extravasation into bladder tissue, whereas micturition voiding pressure, voided volume, post-void residual, volume threshold, intercontraction interval, bladder compliance, and volume threshold to elicit non-voiding contractions as well as urothelium thickness were significantly decreased in CYP-injected rats. Administration of GSK 269962 normalized the abovementioned CYP injection-induced changes. Inhibition of ROCK was found to ameliorate CYP-induced detrusor overactivity and bladder inflammation. Our data indicate uroprotective effects following ROCK inhibition, which further suggests that this strategy may become an interesting pharmacological tool to prevent urinary adverse effects in patients treated with chemotherapy using CYP.

## Introduction

Hemorrhagic cystitis (HC) is a life-threatening condition defined by lower urinary tract symptoms that include hematuria and bladder detrusor overactivity. HC occurs in 10 to 40% of patients receiving high-dose cyclophosphamide (CYP) (Emadi et al. 2009; Watson and Notley 1973), which is one of the most widely used chemotherapy drugs. The severity of HC induced by CYP ranges from mild to severe and may require a variety of procedures like hydration, clot extraction via cystoscopy, continuous bladder irrigation, or cystectomy (Matz and Hsieh 2017); however, despite those treatment options, can be fatal (Ebiloglu et al. 2016). Therefore, its prevention is a great challenge. Administration of a sulfhydryl compound MESNA and hyperhydration are two most frequently used methods for the prevention of HC in patients treated with CYP, but they are not always effective and cannot be used in all patient populations (Payne et al. 2013). Hence, there is a need for novel agents that could be useful to prevent adverse urologic effects during CYP therapy.

Increasing evidence suggests that Rho kinase (ROCK) is an interesting target for the treatment of bladder overactivity. ROCK belongs to the family of serine/threonine AGC (cyclic AMP-dependent protein kinase A (PKA)/protein kinase G (PKG)/protein kinase C (PKC)) kinases. Two major isoforms ROCK1 and ROCK2 have been identified and both are expressed in the urinary bladder (Wibberley et al. 2003). ROCK phosphorylates substrates such as LIM kinase, myosin light chain (MLC) phosphatase, and directly MLC, thus, plays a key role in smooth muscle contraction, including those of the urinary bladder detrusor (Wróbel et al. 2008). As an effector protein of the small GTPase Rho, it is involved in the mechanism of action of antimuscarinic drugs, which are approved for the treatment of overactive bladder (OAB) and which clinical efficacy is driven by occupation at muscarinic M_3_ receptors, since Rho protein can be activated following M_3_ activation (Hegde 2006). Moreover, as ROCK inhibitors were found to be active in several animal models of OAB, direct inhibition of ROCK has been proposed as a novel strategy for the treatment of OAB (Dimitropoulos and Gravas 2016).

In addition to its role in smooth muscle contraction, growing evidence indicates the involvement of ROCK in inflammatory processes (LoGrasso and Feng 2009). Studies on the pathophysiology of CYP-induced HC have shown that it is a non-microbial inflammation, initiated by the contact of CYP liver metabolite, acrolein, with urothelium, in which proinflammatory cytokines (such as tumor necrosis factor-α (TNF-α) and interleukin-1β (IL-1β)) and transcription factors (such as nuclear factor kappa B (NF-κB)) participate (Dobrek and Thor 2012; Korkmaz et al. 2007). One of the crucial mechanisms of inflammatory events after acrolein enters the uroepithelial cells is the effect on NF-κB (Korkmaz et al. 2007). ROCK can activate NF-κB (Segain et al. 2003); therefore, its inhibition seems to be a possible therapeutic strategy for the treatment of CYP-induced HC.

We have previously demonstrated that acute treatment with a ROCK inhibitor, GSK 269962, reversed acetic acid-induced and ovariectomy-induced changes in cystometric parameters (Wróbel and Rechberger 2015; Wróbel and Rechberger 2016a). Furthermore, a combined treatment of low, ineffective doses of GSK 269962 and low, ineffective doses of antimuscarinic drug, solifenacin, triggered a reversal of ovariectomy-induced cystometric alterations (Wróbel and Rechberger 2016a). Considering the potential influence of ROCK inhibition on bladder overactivity and inflammatory cascade, here, we tested whether GSK 269962 would rescue cystometric and inflammatory changes in a model of cystitis induced by CYP injection, which is widely used for investigation of the pathophysiology, prevention, and treatment of HC as well as a model for bladder inflammation and OAB (Juszczak et al. 2010; Lee et al. 2014).

## Materials and methods

### Animals

All procedures were conducted in accordance with the European Communities Council Directive of 22 September 2010 (2010/63/EU) and Polish legislation acts concerning animal experimentations. The experimental procedures and protocols were approved by the First Local Ethics Committee at the Medical University of Lublin. Sixty female Wistar rats were used in the study and were randomly divided into four groups of 15 animals each. Rats were placed individually in metabolic cages (3700M071, Tecniplast, West Chester, PA, USA) with free access to food and water. All experimental procedures were carried out between 8 a.m. and 1 p.m.

All the surgical procedures were performed under anesthesia with intraperitoneal (i.p.) injection of 75 mg/kg of ketamine hydrochloride (Ketanest, Pfizer) and 15 mg/kg of xylazine (Sedazin, Biowet, Puławy, Poland). It was reported that ketamine in combination with xylazine does not abolish the micturition reflex in female rats (Cannon and Damaser 2001).

### Drugs

GSK 269962 (N-[3-[[2-(4-Amino-1,2,5-oxadiazol-3-yl)-1-ethyl-1H-imidazo[4,5-c]pyridin-6-yl]oxy]phenyl]-4-[2-(4-orpholinyl)ethoxy]benzamide, Tocris), a potent ROCK inhibitor (IC50 values: 1.6 and 4 nM for ROCK1 and ROCK2, respectively) was dissolved in DMSO and administered intravenously (i.v.) at a daily dose of 30 mg/kg. CYP (Endoxan, Baxter Deutschland GmbH, Unterschleißheim, Germany) was diluted with saline (0.9% NaCl) and administered i.p. at a single dose of 200 mg/kg. The control rats received volume-matched saline i.p. The doses of the administered agents were chosen based on the literature data and were confirmed/adjusted in our laboratory in preliminary experiments (Hidalgo-Lucas et al. 2016; Juszczak et al. 2010; Kobayashi et al. 2009; Wróbel and Rechberger 2015; Wróbel and Rechberger 2016a; Wróbel and Rechberger 2016b; Wróbel and Rechberger 2016c).

### Study design

The rats received GSK 269962 or vehicle for 7 days. On day 7, 1 h after the last injection of GSK 269962, the animals received CYP or a corresponding volume of saline. On day 8, the surgical procedures were performed. On day 9, 48 h after CYP injection, cystometric studies with physiological saline were performed in conscious unrestrained rats. Following cystometric studies, bladder edema and urothelium thickness were measured.

### Surgical procedures

The surgical procedures have been previously described in detail (Wróbel et al. 2015). In brief, the abdominal wall was opened through an approximately 10-mm vertical midline incision. A double lumen catheter was inserted through the apex of the bladder dome and fixed with 6–0 suture. The inner and outer diameters of the catheter were 0.28 and 0.61 mm, respectively. In the same session, the right femoral vein was catheterised through an inguinal approach. The catheters were tunneled subcutaneously (s.c.) and exteriorized in the retroscapular area, where they were connected with a plastic adapter, to avoid the risk of removal by the animal. The abdomen was closed in multiple layers. Anatomic layers were closed using 4/0 catgut sutures. The free ends of catheters were sealed with silk ligatures. The animals were injected s.c. with 100 mg of cefazolin sodium hydrate (Biofazolin, Sandoz, Holzkirchen, Germany) to prevent urinary tract infection.

### Conscious cystometry

Cystometric investigations were performed in conscious unrestrained rats 1 day after surgical procedures. The bladder catheter was connected via a three-way stopcock to a pressure transducer (FT03, Grass Technologies, West Warwick, RI, USA) and to a microinjection pump (CMA 100, CMA Microdialysis AB, Kista, Sweden). Conscious cystometry was performed by slowly filling the bladder with physiological saline at a constant rate 0.05 ml/min to elicit repetitive voiding. Micturition volumes were measured by means of a fluid collector attached to a force displacement transducer (FT03C). Both transducers were connected to a polygraph (7 DAG, Grass Technologies, West Warwick, RI, USA). Cystometry profiles and micturition volumes were recorded continuously on a Grass polygraph (Model 7E, Grass Technologies, West Warwick, RI, USA) and were determined graphically. The data were analyzed using a sampling rate of 10 samples. The measurements in each animal represent the average of five bladder micturition cycles after obtaining repetitive voiding.

The following cystometric parameters were recorded: basal pressure (BP, cm H_2_O), threshold pressure (TP, cm H_2_O), micturition voiding pressure (MVP, cm H_2_O), voided volume (VV, ml), post-void residual (PVR, ml), volume threshold (VT, ml), voiding efficiency (VE, %), intercontraction interval (ICI, s), bladder contraction duration (BCD, s), relaxation time (RT, s), bladder compliance (BC, ml/cm H_2_O), detrusor overactivity index (DOI, cm H_2_O/ml), non-voiding contractions amplitude (ANVC, cm H_2_O), non-voiding contractions frequency (FNVC, times/filling phase), and volume threshold to elicit NVC (VTNVC, %).

### Bladder edema measurement

Bladder edema was quantified by the determination of vesical vascular permeability, which was measured by the Evans Blue dye leakage technique. Evans Blue at a dose of 50 mg/kg was injected i.v. via a polyethylene catheter inserted into the right femoral vein 30 min before the animals were sacrificed. The bladders were excised, weighted, sliced longitudinally, and placed in 1 ml of formamide solution at 56 °C for 24 h. Formamide absorbance was measured at 620 nM and was compared to the standard curve. The results are presented as nanogram of Evans Blue per milligram of the bladder.

### Urothelium thickness measurement

The image analyzer computer system Leica Qwin 500 Image Analyzer (Leica Imaging Systems Ltd., Cambridge, England) was used to evaluate the urothelium thickness in micrometer using the interactive measure menu and hematoxylin and eosin-stained sections. A mean of 15 readings was estimated from five serial sections from slides of each animal in each group using low magnification (×10).

### Statistical analysis

The obtained data were assessed by the one-way analysis of variance (ANOVA) followed by Tukey’s post hoc test (Statistica, v. 10, StatSoft, Inc., Tulsa, OK, USA). All results are presented as the means ± standard error of the mean (SEM). *p* < 0.05 was considered as a statistically significant difference.

## Results

### The effects of pre-treatment with GSK 269962 on CYP-induced changes in cystometric parameters

One-way ANOVA demonstrated significant changes in cystometric parameters: BP (*F*(3,59) = 12.96, *p* < 0.0001), TP (*F*(3,56) = 86.85, *p* < 0.0001), MVP (*F*(3,56) = 13.83, *p* < 0.0001), VV (*F*(3,56) = 21.31, *p* < 0.0001), PVR (*F*(3,56) = 14.64, *p* < 0.0001), VT (*F*(3,56) = 5.645, *p* = 0.0019), ICI (*F*(3,56) = 66.39, *p* < 0.0001), BCD (*F*(3,56) = 21.13, *p* < 0.0001), RT (*F*(3,56) = 26.05, *p* < 0.0001), BC (*F*(3,56) = 39.59, *p* < 0.0001), DOI (*F*(3,56) = 31.10, *p* < 0.0001), ANVC (*F*(3,56) = 53.20, *p* < 0.0001), FNVC (*F*(3,56) = 132.4, *p* < 0.0001), VTNVC (*F*(3,56) = 42.12, *p* < 0.0001) between the examined groups, whereas VE parameter did not differ significantly between the examined groups. The following parameters were significantly increased in CYP-injected rats: BP, TP, BCD, RT, DOI, ANVC, FNVC, whereas MVP, VV, PVR, VT, ICI, BC, VTNVC parameters were significantly decreased in those rats, compared to control animals. Administration of GSK 269962 to animals that received saline instead of CYP did not significantly affect the values of the cystometric parameters, compared to control rats. Administration of GSK 269962 to animals that received CYP induced significant differences in the parameters: BP, TP, MVP, VV, PVR, VT, ICI, BCD, RT, BC, DOI, ANVC, FNVC, and VTNVC, compared to CYP group. Administration of GSK 269962 to animals that received CYP induced also significant differences in the parameters: ICI, RT, BC, DOI, ANVC, FNVC and VTNVC, compared to GSK 269962 group (Table 1).

**Table 1:** The effects of pre-treatment with GSK 269962 (30 mg/kg i.v., once daily for 7 consecutive days) on cyclophosphamide (CYP, 200 mg/kg, i.p., single dose) induced changes in cystometric parameters

	BP (cm H_2_O)	TP (cm H_2_O)	MVP (cm H_2_O)	VV (ml)	PVR (ml)	VT (ml)	VE (%)	ICI (s)	BCD (s)	RT (s)	BC (ml/cm H_2_O)	DOI (cm H_2_O/ml)	ANVC (cm H_2_O)	FNVC (times/ filling phase)	VTNC %
Control	2.973 ± 0.180	7.560 ± 0.319	32.37 ± 1.128	0.776 ± 0.031	0.074 ± 0.003	0.617 ± 0.025	90.53 ± 1.050	932.5 ± 21.81	30.00 ± 0.899	20.53 ± 0.425	0.184 ± 0.008	122.7 ± 3.309	2.347 ± 0.085	0.617 ± 0.041	49.98 ± 1.608
GSK 269962	3.427 ± 0.159	7.487 ± 0.372	33.17 ± 1.497	0.664 ± 0.041	0.065 ± 0.004	0.607 ± 0.038	90.00 ± 1.320	1001 ± 40.82	32.33 ± 1.286	22.37 ± 0.787	0.187 ± 0.005	108.7 ± 3.616	2.280 ± 0.085	0.460 ± 0.042	48.47 ± 1.279
CYP	5.207 ± 0.470***	15.48 ± 0.573***	23.52 ± 0.926***	0.421 ± 0.024***	0.040 ± 0.003***	0.452 ± 0.018**	87.93 ± 1.596	381.7 ± 27.82***	41.80 ± 1.083***	31.17 ± 1.275***	0.082 ± 0.007***	253.8 ± 21.25***	6.243 ± 0.432***	9.350 ± 0.643***	29.41 ± 1.049***
CYP + GSK 269962	3.373 ± 0.162^###^	8.00 ± 0.364^###^	30.53 ± 1.101^###^	0.633 ± 0.029^###^	0.067 ± 0.004^###^	0.605 ± 0.043^#^	89.13 ± 1.496	701.1 ± 42.33^###, $$$^	36.33 ± 1.190^##^	26.51 ± 1.005^##, $^	0.117 ± 0.010^#, $$$^	165.8 ± 8.620^###, $$^	3.809 ± 0.239^###, $$$^	1.929 ± 0.348^###, $^	38.69 ± 1.837^###, $$$^

Data were analyzed by the one-way analysis of variance (ANOVA) followed by Tukey’s post hoc test; values are expressed as the mean ± SEM. ***p < 0.001, **p < 0.01 vs control; ^###^ *p* < 0.001, ^##^ *p* < 0.01, ^#^ *p* < 0.05 vs CYP; ^$$$^ *p* < 0.001, ^$$^ *p* < 0.01, ^$^ *p* < 0.05 vs GSK 269962. One-way ANOVA demonstrated significant changes in: BP (*F*(3,59) = 12.96, *p* < 0.0001), TP (*F*(3,56) = 86.85, *p* < 0.0001), MVP (*F*(3,56) = 13.83, *p* < 0.0001), VV (*F*(3,56) = 21.31, *p* < 0.0001), PVR (*F*(3,56) = 14.64, *p* < 0.0001), VT (*F*(3,56) = 5.645, *p* = 0.0019), ICI (*F*(3,56) = 66.39, *p* < 0.0001), BCD (*F*(3,56) = 21.13, *p* < 0.0001), RT (*F*(3,56) = 26.05, *p* < 0.0001), BC (*F*(3,56) = 39.59, *p* < 0.0001), DOI (*F*(3,56) = 31.10, *p* < 0.0001), ANVC (*F*(3,56) = 53.20, *p* < 0.0001), FNVC (*F*(3,56) = 132.4, *p* < 0.0001), VTNVC (*F*(3,56) = 42.12, *p* < 0.0001), whereas VE did not differ significantly between the examined groups. The following parameters were significantly increased in CYP group: BP (*p* < 0.001), TP (*p* < 0.001), BCD (*p* < 0.001), RT (*p* < 0.001), DOI (*p* < 0.001), ANVC (*p* < 0.001), and FNVC (*p* < 0.001), whereas MVP (*p* < 0.001), VV (*p* < 0.001), PVR (*p* < 0.001), VT (*p* < 0.01), ICI (*p* < 0.001), BC (*p* < 0.001), and VTNVC (*p* < 0.001) parameters were significantly decreased, compared to the control group. Administration of GSK 269962 to animals that received saline instead of CYP (GSK 269962 group) did not significantly affect the values of the cystometric parameters, compared to the control group. Administration of GSK 269962 to animals that received CYP (CYP + GSK 269962 group) induced a significant increase in MVP (*p* < 0.001), VV (*p* < 0.001), PVR (*p* < 0.001), VT (*p* < 0.05), ICI (*p* < 0.001), BC (*p* < 0.05), and VTNVC (*p* < 0.001), and a decrease in BP (*p* < 0.001), TP (*p* < 0.001), BCD (*p* < 0.01), RT (*p* < 0.01), DOI (*p* < 0.001), ANVC (*p* < 0.001), and FNVC (*p* < 0.001), compared to CYP group. Administration of GSK 269962 to animals that received CYP (CYP + GSK 269962 group) induced also a significant increase in: RT (*p* < 0.05), DOI (*p* < 0.01), ANVC (*p* < 0.001), FNVC (*p* < 0.05) and a decrease in ICI (*p* < 0.001), BC (*p* < 0.001), and VTNVC (*p* < 0.001), compared to GSK 269962 group

*BP* basal pressure (cm H_2_O), *TP* threshold pressure (cm H_2_O), *MVP* micturition voiding pressure (cm H_2_O), *VV* voided volume (ml), *PVR* post-void residual (ml), *VT* volume threshold (ml), *VE* voiding efficiency (%), *ICI* intercontraction interval (s), *BCD* bladder contraction duration (s), *RT* relaxation time (s), *BC* bladder compliance (ml/cm H_2_O), *DOI* detrusor overactivity index (cm H_2_O/ml), *ANVC* non-voiding contractions amplitude (cm H_2_O), *FNVC* non-voiding contractions frequency (times/filling phase), *VTNVC* volume threshold to elicit NVC (%)

### The effects of pre-treatment with GSK 269962 on CYP-induced changes in Evans Blue extravasation and urothelium thickness

**Fig. 1:**
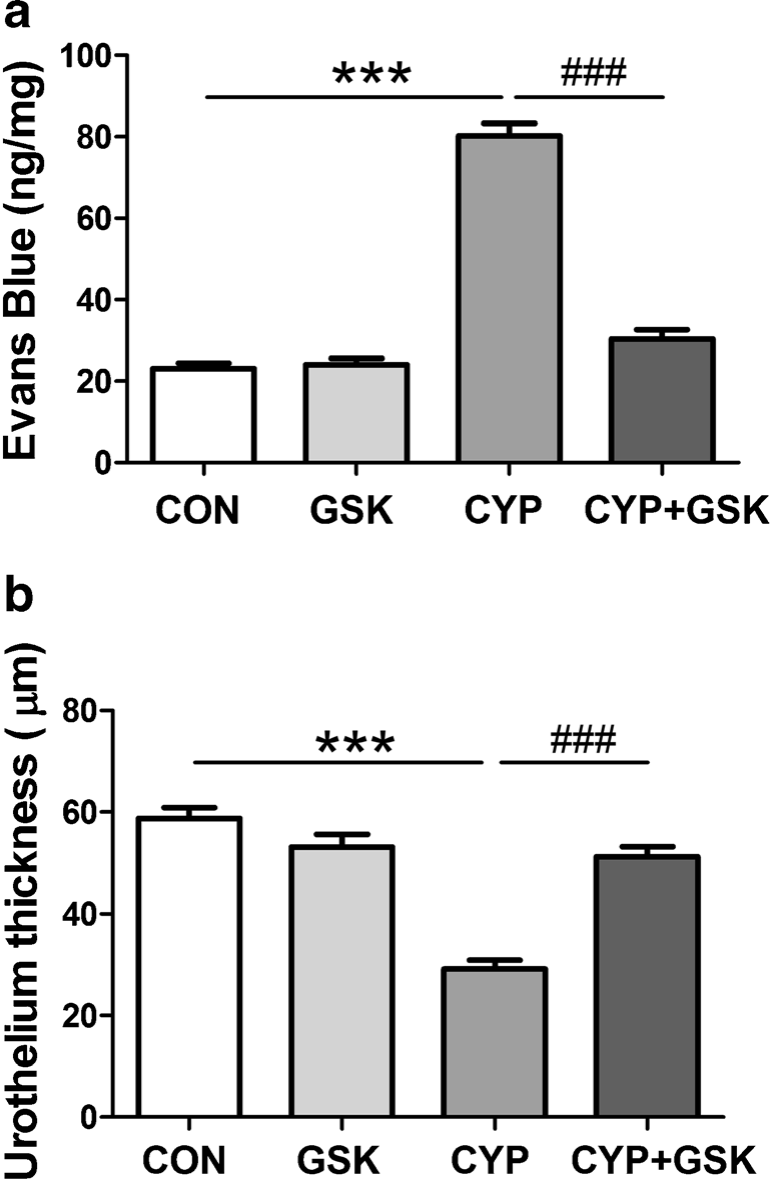
The effects of pre-treatment with GSK 269962 (GSK) (30 mg/kg i.v., once daily for 7 consecutive days) on cyclophosphamide (CYP, 200 mg/kg, i.p., single dose) induced changes in Evans blue extravasation **(a)** and urothelium thickness **(b)**. Data were analyzed by the one-way analysis of variance (ANOVA) followed by Tukey’s post hoc test; values are expressed as the mean ± SEM. ***p < 0.001 vs control (CON), ^###^ *p* < 0.001 vs CYP

One-way ANOVA demonstrated significant changes in Evans Blue extravasation into bladder tissue (*F*(3,56) = 152.9, *p* < 0.0001) (Fig. 1a) and urothelium thickness (*F*(3,56) = 38.53, *p* < 0.0001) (Fig. 1b). CYP injection significantly increased Evans Blue extravasation and decreased urothelium thickness in rats, compared to control animals. Administration of GSK 269962 to animals that received saline instead of CYP did not significantly affect Evans Blue extravasation or urothelium thickness, compared to control rats. Administration of GSK 269962 to animals that received CYP significantly decreased Evans Blue extravasation and increased urothelium thickness, compared to CYP group, but did not affect these parameters compared to GSK 269962 group (Fig. 1a, b).

## Discussion

Chemotherapy with oxazaphosphorines, such as CYP, is often limited by urotoxicity, of which HC is a potentially life-threatening condition. As HC may require inpatient management and major procedures, which are not always effective (Payne et al. 2013), its prevention is preferred over treatment. Because CYP-induced HC is an inflammatory process (Korkmaz et al. 2007), compounds that possess anti-inflammatory properties seem worth taking into account while searching for agents that could be beneficial for reducing adverse effects during CYP therapy. Here, we found that ROCK inhibitor, GSK 269962, ameliorated changes: increased plasma protein extravasation and decreased urothelium thickness, which were observed after CYP injection. Our study shows anti-inflammatory activity of GSK 269962 in the model of acute, chemical, CYP-induced cystitis.

Other ROCK inhibitor, hydroxyfasudil, was found to exert anti-inflammatory effect in the model of HCl-induced chemical cystitis (Shimizu et al. 2013). Hydroxyfasudil reduced the severity of histological changes associated with HCl administration, such as epithelial denudation, submucosal edema, inflammatory cell infiltrate, tissue granulation, and vasodilation (Shimizu et al. 2013). Moreover, hydroxyfasudil was shown to exhibit anti-inflammatory properties in the model of protamine sulfate-induced cystitis, as demonstrated by histopathological assessment (Akin et al. 2015). Thus, our and other findings support the role of ROCK inhibition in reducing inflammation of the urinary bladder.

Data have indicated the involvement of ROCK in the pathophysiology of bladder dysfunction. ROCK expression at the gene and protein level as well as its activity were increased in bladder smooth muscle of hepatic-specific insulin receptor substrate 1 and 2 double knockout mice, which develop type 2 diabetes and bladder dysfunction; furthermore, ROCK expression and activity correlated with hyper- and hypoactivity states of the bladder (Wang et al. 2012). Expression of ROCK1 and ROCK2 at the mRNA level was also increased in the bladder of rats in the model of HCl-induced cystitis (Shimizu et al. 2013).

In the present study, we found that GSK 269962 ameliorated CYP injection-induced changes in all examined cystometric parameters. We have previously shown that acute treatment with GSK 269962 normalized acetic acid-induced and ovariectomy-induced cystometric changes (Wróbel and Rechberger 2015; Wróbel and Rechberger 2016a). Furthermore, a combined treatment of low doses of GSK 269962 and low doses of antimuscarinic drug, solifenacin, both ineffective as monotherapies, triggered a reversal in cystometric alterations caused by ovariectomy (Wróbel and Rechberger 2016a). Hydroxyfasudil was also shown to ameliorate CYP injection-induced detrusor overactivity (Masago et al. 2009). Considering the effects of ROCK inhibition on cystometric parameters as well as the involvement of ROCK in the pathophysiology of bladder dysfunction, our data further support the hypothesis that inhibition of this kinase might be a new therapeutic strategy for bladder overactivity.

Uroprotective agent currently used in patients undergoing CYP therapy, namely MESNA, is administered in divided doses—prior and after CYP administration. This treatment schedule highlights the need for administration of uroprotective agent prior to CYP, what influenced our study design. We focused on pre-treatment with GSK 269962 and used the dose which was effective in our models of acetic acid-induced and ovariectomy-induced-detrusor overactivity (Wróbel and Rechberger 2015; Wróbel and Rechberger 2016a).

It has been proposed that CYP-induced HC is initiated by direct contact of its metabolite, acrolein, with urothelium, which causes edema, ulceration, neovascularization, hemorrhage, and necrosis (Lee et al. 2014). One of the most important mechanisms of inflammatory events after acrolein enters into the uroepithelial cells is the effect on NF-κB. (Korkmaz et al. 2007). ROCK is one of the factors activating NF-κB, while its blockade was found to prevent inflammation via NF-κB inhibition (Segain et al. 2003). Thus, beneficial effects of GSK 269962 in ameliorating inflammation might result from inhibition of NF-κB.

Other pivotal mediators of inflammatory reaction induced by CYP are proinflammatory cytokines: TNF-α and IL-1β. It was shown that pre-treatment with anti-TNF-α or anti-IL-1β serum diminished mucosal erosion, hemorrhage, edema, leukocyte migration, and ulceration in CYP-injected mice (Gomes et al. 1995). GSK 269962 was found to suppress generation of TNF-α in lipopolysaccharide-stimulated monocytes (Doe et al. 2007). Other ROCK inhibitor, Y27632, was found to block the increase in TNF-α and IL-1β release upon lipopolysaccharide injection in vivo (Wang et al. 2012). Therefore, the possible mechanism by which GSK 269962 diminished CYP-induced inflammation may be associated with inhibition of TNF-α and/or IL-1β. It should be noted that TNF-α and IL-1β activate NF-κB through degradation of an inhibitory protein to which NF-κB is connected in the cytoplasm. This allows NF-κB enter the nucleus and subsequently promote transcription of genes for proinflammatory cytokines, such as TNF-α and IL-1β (Karin and Ben-Neriah 2000; Lawrence 2009). Thus, the possible inhibition of TNF-α and/or IL-1β by GSK 269962 could also lead to inhibition of NF-κB.

Another potential mechanism of beneficial effects of ROCK inhibition on inflammatory changes in CYP-induced cystitis may be due to the role of ROCK in leukocyte extravasation. Migration of leukocytes across the endothelial layer is a hallmark of inflammation. Activation of a ROCK–MLC kinase pathway stimulates endothelial, actomyosin-based contractility, whereas inhibition of this pathway reduces leukocytes transendothelial migration (Hordijk 2016). However, direct mechanisms by which GSK 269962 exerts anti-inflammatory effect in a model of CYP-induced cystitis need further examination.

## Conclusions

Considering the effects of GSK 269962 on inflammatory and cystometric parameters, inhibition of ROCK may be a new pharmacological option for the treatment of bladder inflammation and detrusor overactivity. Because GSK 269962 ameliorated CYP-induced bladder inflammation and overactivity, our data indicate uroprotective effects following ROCK inhibition, which further suggests that this strategy may become a pharmacological tool to prevent urinary adverse effects in patients treated with chemotherapy using CYP. ROCK inhibitors may represent a novel class of uroprotective agents, targeting inflammatory basis of HC, i.e., pathogenesis-based, which beneficial role in preventing/reducing CYP-induced HC may, at least partly, result from anti-inflammatory properties.
